# Pancreatic Cystic Neoplasm Risk Among Individuals With Diabetes

**DOI:** 10.1001/jamanetworkopen.2025.56951

**Published:** 2026-02-13

**Authors:** In Rae Cho, Sung Hoon Chang, Sang Hyub Lee, Kyung-Do Han, Kwang Hyun Chung, Min Woo Lee, Jin Ho Choi, Woo Hyun Paik, Ji Kon Ryu

**Affiliations:** 1 Department of Internal Medicine and Liver Research Institute, Seoul National University College of Medicine, Seoul, Korea; 2 Department of Internal Medicine, Kangwon National University School of Medicine, Chuncheon, Korea; 3 Department of Statistics and Actuarial Science, Soongsil University, Seoul, Republic of Korea; 4 Department of Internal Medicine, Soonchunhyang University Seoul Hospital, Seoul, Republic of Korea

## Abstract

**Importance:**

With advancements in imaging technology and more frequent health evaluations, the incidence and prevalence of pancreatic cysts have gradually increased. Certain types of pancreatic cystic neoplasms are precancerous lesions associated with an increased risk of pancreatic cancer. Hence, identifying risk factors and preventing their occurrence are crucial. Nonetheless, population-based research on modifiable risk factors remains lacking.

**Objective:**

To investigate the association of diabetes and related factors with risk of developing pancreatic cysts.

**Design, Setting, and Participants:**

This population-based cohort study included adults (aged ≥20 years) who underwent health examinations in 2009 through medical institutions designated by the Korean National Health Insurance Service. Participants were followed up until December 31, 2020. Data were analyzed from March 23, 2023, to February 8, 2024.

**Exposure:**

All participants were categorized according to diabetes status as having normoglycemia, impaired fasting glucose, shorter diabetes duration (<5 years), or longer diabetes duration (≥5 years). Demographic characteristics, lifestyle factors, and comorbidities at the time of health examinations were investigated.

**Main Outcomes and Measures:**

Adjusted hazard ratios (AHRs) for pancreatic cyst occurrence for each diabetes status group were estimated using Cox proportional hazards regression models, adjusting for potential confounders.

**Results:**

Among the entire study population of 3 856 676 adults (mean [SD] age, 47.1 [14.0] years; 54.5% male), 330 138 (8.6%) had diabetes. The median observation period was 10.3 (IQR, 10.1-10.6) years. A total of 31 877 patients (0.8%) developed pancreatic cysts during the observation period. Compared with individuals with normoglycemia, AHRs for the development of pancreatic cysts were 1.06 (95% CI, 1.03-1.08) for those with impaired fasting glucose, 1.23 (1.18-1.28) for those with a shorter diabetes duration, and 1.37 (1.31-1.44) for those with a longer diabetes duration. Subgroup analyses showed higher AHRs for pancreatic cyst occurrence associated with diabetes among individuals younger than 60 years (AHR, 1.34 [95% CI, 1.27-1.40]), males (AHR, 1.32 [95% CI, 1.26-1.38]), and current smokers (AHR, 1.40 [95% CI, 1.30-1.51]) with diabetes compared with patients 60 years or older (AHR, 1.21 [95% CI, 1.16-1.27]), females (AHR, 1.20 [95% CI, 1.15-1.26]), never smokers (AHR, 1.22 [95% CI, 1.18-1.28]), and former smokers (AHR, 1.25 [95% CI, 1.16-1.35]) with diabetes.

**Conclusions and Relevance:**

In this cohort study of 3 856 676 Korean adults, longer diabetes duration was associated with an increased risk of pancreatic cysts. The risk of pancreatic cyst occurrence was higher among younger male individuals with diabetes compared with their counterparts. Smoking cessation was associated with a lower risk of pancreatic cysts. Further studies incorporating imaging and longitudinal data are needed to clarify the clinical significance of pancreatic cysts in individuals with diabetes.

## Key Points

**Question:**

Is diabetes associated with an increased risk of developing pancreatic cystic neoplasms (PCNs)?

**Findings:**

In this cohort study of 3 856 676 individuals in Korea followed up for a median of 10 years, longer diabetes duration (≥5 years) was associated with an increased risk of PCNs after adjustment for major confounding factors. This risk was particularly elevated in individuals aged younger than 60 years with diabetes, males with diabetes, and current smokers with diabetes.

**Meaning:**

These findings suggest that diabetes may be a factor in the development of PCNs and highlight the potential need for targeted surveillance in individuals with diabetes.

## Teaser

This cohort study examines the association between diabetes status and risk of pancreatic cystic neoplasm development among Korean adults.

## Introduction

Pancreatic cysts are a heterogeneous group of lesions ranging from nonneoplastic pseudocysts and pancreatic cystic neoplasms (PCNs) to cystic degeneration of solid tumors.^1^ The incidence and prevalence of pancreatic cysts have increased with an aging population and the widespread availability of cross-sectional imaging.^2,3^ While most pancreatic cysts are found incidentally and exhibit an indolent clinical course, a subset of PCNs that includes mucinous cystic neoplasms (MCNs) and intraductal papillary mucinous neoplasms (IPMNs) has malignant potential.^4^

After a pancreatic cyst is identified, the follow-up strategy varies depending on its subtype. For pancreatic cysts with unequivocally benign features such as nonneoplastic pseudocysts and serous cystadenomas (SCAs), further evaluation is not necessary and minimal surveillance is sufficient. In contrast, for PCNs with malignant potential, clinical guidelines recommend continuous surveillance for patients who are medically fit for surgery with sufficient life expectancy, unless long-term stability of small cysts without worrisome features has been confirmed.^2,5^ Unspecified cysts that cannot be sufficiently characterized through imaging studies are presumed to be mucinous cysts.^4^ Therefore, a large number of individuals with PCNs undergo regular imaging tests, leading to unwarranted anxiety and imposing a substantial burden on the health care system.^6^

Considering these practical challenges, identifying risk factors for PCN development and preventing its occurrence are clinically important. Advanced age, hereditary cancer syndrome, family history of pancreatic cancer, and genetic variants (eg, *KRAS* or *GNAS*) have been identified by previous studies as risk factors for IPMNs.^7,8,9^ Nevertheless, these risk factors are inevitable and unavoidable, and research on modifiable risk factors remains insufficient. Thus, specific measures for reducing PCN occurrence are limited.

Hyperglycemia induces oxidative stress and inflammation in the body and leads to various complications.^10,11^ Diabetes is known to be associated with the risk of pancreatic cancer.^12^ Nonetheless, investigations of the association between diabetes and PCN occurrence are lacking. We assumed that diabetes is related to PCN occurrence based on the fact that repetitive and chronic inflammation induced by hyperglycemia can lead to tumorigenesis in susceptible individuals.

Previous nationwide population-based studies including Korean adults have reported an epidemiologic correlation between diabetes and pancreatic diseases.^13^ This study aimed to investigate the risk of PCNs based on diabetes status and to explore whether modifiable factors are associated with PCN development in patients with diabetes.

## Methods

This cohort study was approved by the Seoul National University Hospital Institutional Review Board and was conducted in accordance with the principles embodied in the Declaration of Helsinki.^14^ The need for informed consent was waived owing to the retrospective nature of the study and the use of anonymized data. The study followed the Strengthening the Reporting of Observational Studies in Epidemiology (STROBE) reporting guideline.

### Data Source

This nationwide population-based study used data from the Korean National Health Insurance Service (NHIS) database to investigate the association between pancreatic cyst development and diabetes. The NHIS is a single health insurance system administered by the Korean government that covers 97% of the population except for 3% of medical protection beneficiaries with low income who receive aid through the Medical Aid Program.^15^ The NHIS has established a public medical big data platform based on data accumulated for the purpose of claims by medical institutions.^16^ Individuals’ use of medical facilities, prescription details, diagnostic codes configured in the form of *International Statistical Classification of Diseases and Related Health Problems, Tenth Revision* (*ICD-10*) codes, and income decile are recorded in the NHIS database. This database, which covers nearly the entire Korean population, is closest to clinical data and can be used in research because of its anonymization and deidentification.^17^ The NHIS provides a general health examination program for all beneficiaries aged 20 years or older and all employees of any age. This health examination program involves anthropometric measurements, use of a self-administered questionnaire on medical history or health-related behaviors, and laboratory testing.^13^

### Study Participants

Patients who underwent a health examination provided by the NHIS in 2009 were selected through a standardization process and comprised the study cohort. Participants who met the exclusion criteria described next for identification of pancreatic cysts, diabetes status, and comorbidities, individuals with a history of pancreatic cysts from 2002 until the time of health check-up (washout), participants diagnosed with a pancreatic cyst within 1 year of their health check-up (1-year lag period), and those with missing data were excluded from the analysis. All participants were followed up until December 31, 2020. Data were analyzed from March 23, 2023, to February 8, 2024.

### Identification of Pancreatic Cysts, Diabetes Status, and Comorbidities

Incident pancreatic cysts were identified based on *ICD-10* codes for both pancreatic cysts (K86.2) and PCNs (D13.6 and D37.7) in the NHIS claim database.^18^ To refine our case definition for PCNs and minimize the inclusion of nonneoplastic cysts, which are typically caused by acute or chronic pancreatitis, both preexisting pancreatic cysts and participants with *ICD-10* codes K85.x (acute pancreatitis), K86.0 (alcoholic pancreatitis), K86.1 (chronic pancreatitis), K86.3 (pancreatic pseudocyst), or K86.8 (pancreatic fluid collection) diagnosed prior to the index date (health examination) or even after baseline but before PCN occurrence were excluded from the analysis. Because the NHIS database does not contain information on imaging modalities, the diagnostic method for pancreatic cysts could not be directly determined.

Participant diabetes status and glucose tolerance status were categorized into groups according to *ICD-10* codes for diabetes (E11.x-E14.x), prescription records (oral or injectable antidiabetic medications or both), and fasting glucose measurements during health examination. This definition was based on the consensus of relevant findings used in previous studies.^19,20^ Participants were classified according to glycemic status as having normoglycemia (fasting glucose level <100 mg/dL), impaired fasting glucose (IFG) (fasting glucose level of 100-125 mg/dL), or diabetes based on the American Diabetes Association criteria.^21^ Diabetes duration, defined as shorter (<5 years) or longer (≥5 years), was identified based on the time point from the date of the first antidiabetic medication prescription to the date of the health examination. The health examination date served as the common baseline (time origin) for subsequent follow-up in the Cox regression analyses.

Baseline comorbidities at the time of health examination were identified as hypertension, dyslipidemia, or chronic kidney disease (CKD). Hypertension was defined using *ICD-10* codes (I10.x-I13.x or I15.x) and antihypertensive medication use, systolic/diastolic blood pressure of 140/90 mm Hg or greater, or both. Dyslipidemia was defined using *ICD-10* code E78.x and treatment with lipid-lowering agents, a serum total cholesterol level of 240 mg/dL or greater, or both. CKD was defined as an estimated glomerular filtration rate (eGFR) of less than 60 mL/min/1.73 m^2^ or having at least 1 claim with a special code (V code) indicating the initiation of renal replacement therapy (hemodialysis [V001], peritoneal dialysis [V003], or kidney transplantation [V005]). *ICD-10* codes were also used to define myocardial infarction (I21.x-I22x) and stroke (I63.x-I64.x).

### Clinical Variables and Subgroup Analysis

Clinical variables obtained from the health examination and NHIS database were used as baseline characteristics.^13^ During the health examination, anthropometric measurements (height, weight, and waist circumference), systolic/diastolic blood pressure assessment, self-administered questionnaires, and laboratory tests were performed. Based on the standardized self-reported questionnaires, detailed information about participants’ lifestyles was obtained and classified according to smoking status (never smoker, former smoker, or current smoker), alcohol consumption (none, no consumption; mild to moderate, mean consumption <30 g/d in men or <20 g/d in women; or heavy, mean consumption ≥30 g/d in men or ≥20 g/d in women), and regular physical activity (defined as vigorous-intensity physical activity for ≥20 minutes for ≥3 d/wk or moderate-intensity physical activity for ≥30 minutes for ≥5 d/wk).

Blood samples were collected after overnight fasting, and serum fasting glucose, total cholesterol, high-density lipoprotein cholesterol, low-density lipoprotein cholesterol, triglycerides, aspartate aminotransferase, alanine aminotransferase, γ-glutamyl transferase, and creatinine levels were measured. eGFR was calculated using the Modification of Diet in Renal Disease equation.^22^

### Statistical Analysis

Continuous variables are expressed as means (SDs) or geometric means (95% CIs) for nonnormally distributed variables. Categorical variables are presented as numbers and percentages. Differences among groups were analyzed using the χ^2^ test for categorical variables and the *F* test for continuous variables, with log transformation applied to nonnormally distributed data. The incidence of pancreatic cysts was calculated by dividing the number of incident case patients in each group by the total follow-up period per 1000 person-years. Adjusted hazard ratios (AHRs) and 95% CIs for pancreatic cysts were derived using the Cox proportional hazards regression model. A multivariable-adjusted Cox proportional hazards model was applied: model 1 was adjusted for age, sex, hypertension, dyslipidemia, income status, smoking status, alcohol consumption, regular physical activity, and body mass index. Model 2 was further adjusted for hypertriglyceridemia.

Subgroup analyses were performed to investigate associations between diabetes and PCN occurrence according to age, sex, lifestyle factors, and comorbidities. Each subgroup was defined according to age (<60 or ≥60 years), sex (male or female), smoking status (nonsmoker, former smoker, or current smoker), alcohol consumption (none, mild, or heavy), regular physical activity (participants who did or did not exercise regularly), and hypertriglyceridemia and CKD status. Statistical analyses were performed using SAS, version 9.4 (SAS Institute Inc). Statistical significance was set at 2-sided *P* < .05.

## Results

### Baseline Characteristics

**Figure 1.**
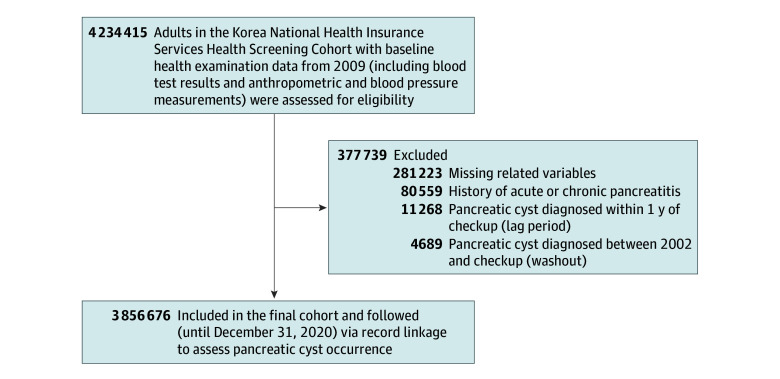
Study Flow Diagram

A total of 4 234 415 individuals underwent a health examination provided by the NHIS in 2009 (Figure 1). Of these, 377 739 were excluded from this study (281 223 had missing data, 80 559 met the exclusion criteria, 11 268 were diagnosed with a pancreatic cyst within the 1-year lag period, and 4689 had a history of pancreatic cysts during the washout period). A total of 3 856 676 individuals thus were included in this analysis. Their mean (SD) age was 47.1 (14.0) years, 45.5% were female and 54.5% were male, and 330 138 (8.6%) had diabetes. The median observation period was 10.3 (IQR, 10.1-10.6) years.

Baseline characteristics of the study population are shown in Table 1. Among the 3 856 676 eligible participants, 68.6% (n = 2 645 696) had normoglycemia, 22.8% (n = 880 842) had IFG, 5.7% (n = 220 004) had a shorter diabetes duration (<5 years), and 2.9% (n = 110 134) had a longer diabetes duration (≥5 years). Patients with diabetes were older and more likely to have obesity. The prevalence of comorbidities (eg, hypertension, dyslipidemia, CKD, myocardial infarction, and stroke) increased with the deterioration of diabetes status and duration. The proportion of current smokers and participants with heavy alcohol consumption was the highest in the group with a shorter diabetes duration. The prevalence of pancreatic cysts was 0.8% in the cohort overall and 1.6% in individuals aged 60 years or older.

**Table 1.** Baseline Characteristics of the Study Population^a^

Variable	Participant diabetes status (N = 3 856 676)				*P* value
	Normoglycemia (n = 2 645 696)	Impaired fasting glucose (n = 880 842)	Diabetes duration (n = 330 138)		
			<5 y (n = 220 004)	≥5 y (n = 110 134)	
Age, mean (SD), y	45.0 (13.8)	49.7 (13.2)	54.9 (12.3)	61.7 (10.0)	<.001
Sex					
Female	1 292 491 (48.9)	335 778 (38.1)	77 190 (35.1)	49 271 (44.7)	<.001
Male	1 353 205 (51.1)	545 064 (61.9)	142 814 (64.9)	60 863 (55.3)	
Smoking status					
Never smoker	1 636 282 (61.8)	481 742 (54.7)	114 505 (52.0)	68 860 (62.5)	<.001
Former smoker	335 564 (12.7)	154 573 (17.5)	40 867 (18.6)	19 892 (18.1)	
Current smoker	673 850 (25.5)	244 527 (27.8)	64 632 (29.4)	21 382 (19.4)	
Alcohol consumption					
None	1 385 102 (52.4)	418 254 (47.5)	113 915 (51.8)	73 278 (66.5)	<.001
Mild	1 062 636 (40.2)	366 342 (41.6)	80 286 (36.5)	29 050 (26.4)	
Heavy	197 958 (7.5)	96 246 (10.9)	25 803 (11.7)	7806 (7.1)	
Regular physical activity					
Yes	452 932 (17.1)	166 907 (18.9)	45 368 (20.6)	26 666 (24.2)	<.001
Comorbidity					
Hypertension	504 082 (19.1)	286 289 (32.5)	113 635 (51.7)	71 330 (64.8)	<.001
Dyslipidemia	351 420 (13.3)	186 542 (21.2)	78 599 (35.7)	48 061 (43.6)	
Hypertriglyceridemia	735 814 (27.8)	365 392 (41.5)	134 725 (61.2)	69 881 (63.4)	
Chronic kidney disease	154 760 (5.8)	69 431 (7.9)	22 341 (10.2)	19 499 (17.7)	
Myocardial infarction	7815 (0.3)	3867 (0.4)	2013 (0.9)	1805 (1.6)	
Stroke	30 638 (1.2)	15 416 (1.8)	7895 (3.6)	8044 (7.3)	
Anthropometric measurements, mean (SD)					
BMI	23.3 (3.2)	24.4 (3.2)	25.2 (3.4)	24.7 (3.1)	<.001
Waist circumference, cm	78.8 (9.0)	82.41 (8.7)	85.6 (8.6)	85.3 (8.2)	
Blood pressure, mm Hg, mean (SD)					
Systolic	120.5 (14.5)	125.8 (15.1)	129.2 (15.8)	129.1 (15.8)	<.001
Diastolic	75.3 (9.8)	78.35 (10.1)	79.8 (10.3)	77.8 (10.0)	
Laboratory findings, mean (SD)					
Fasting glucose, mg/dL	87.5 (7.7)	107.8 (6.6)	146.1 (46.4)	148.7 (54.7)	<.001
Total cholesterol, mg/dL	192.6 (35.5)	201.8 (37.5)	201.2 (42.0)	188.9 (40.7)	
HDL cholesterol, mg/dL	56.8 (27.6)	55.4 (27.7)	52.7 (27.7)	51.92 (30.3)	
LDL cholesterol, mg/dL	112.6 (38.0)	117.6 (38.5)	113.1 (43.1)	106.45 (41.5)	
Triglycerides, mg/dL^b^	104.3 (104.2-104.3)	127.3 (127.1-127.4)	154.3 (153.9-154.7)	138.4 (138.0-138.9)	
AST, U/L^b^	22.7 (22.69-22.71)	24.7 (24.70-24.74)	26.9 (26.8-26.9)	24.2 (24.1-24.2)	
ALT, U/L^b^	20.0 (20.01-20.04)	23.7 (23.62-23.67)	27.8 (27.7-27.9)	23.9 (23.9-24.0)	
γ-GTP, U/L^b^	23.8 (23.7-23.8)	31.4 (31.3-31.4)	40.7 (40.6-40.9)	30.2 (30.1-30.3)	
eGFR, mL/min/1.73 m^2^	89.1 (49.2)	85.1 (35.4)	84.8 (36.8)	80.5 (35.0)	

Abbreviations: ALT, alanine aminotransferase; AST, aspartate aminotransferase; BMI, body mass index (calculated as weight in kilograms divided by height in meters squared); eGFR, estimated glomerular filtration rate; γ-GTP, γ-glutamyl transpeptidase; HDL, high-density lipoprotein; LDL, low-density lipoprotein.

SI conversion factors: To convert fasting glucose to mmol/L, multiply by 0.0555; total, HDL, and LDL cholesterol to mmol/L, multiply by 0.0259; triglycerides to mmol/L, multiply by 0.0113; and AST, ALT, and γ-GTP to μkat/L, multiply by 0.0167.

^a^ Unless indicated otherwise, values are presented as the No. (%) of participants.

^b^ Nonnormally distributed variables are expressed as the geometric mean (95% CI).

### PCN Incidence According to Diabetes Status

**Figure 2.**
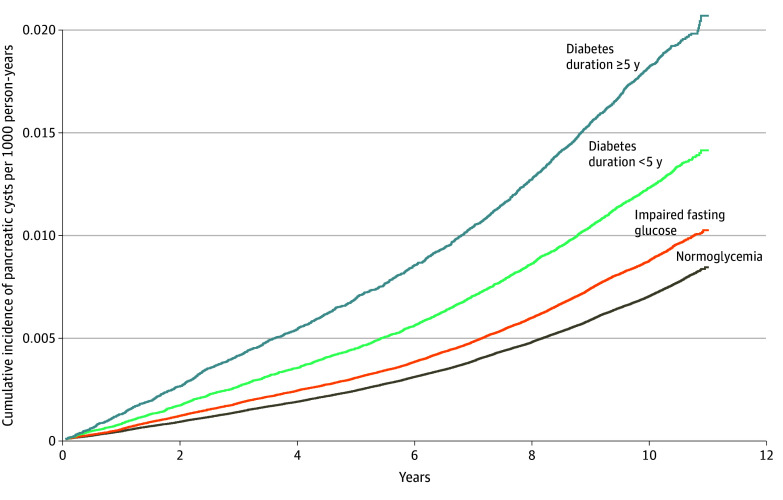
Cumulative Incidence of Pancreatic Cysts by Diabetes Status

During the total observation period of 38 906 756.3 person-years, 31 877 patients with PCNs (0.8%) were identified (Table 2). The overall incidence of PCNs was 0.82 per 1000 person-years in the entire population, with incidences per 1000 person-years of 0.72 (95% CI, 0.71-0.73) in individuals with normoglycemia, 0.89 (95% CI, 0.87-0.91) in those with IFG, 1.25 (95% CI, 1.20-1.30) in those with shorter diabetes duration, and 1.82 (95% CI, 1.74-1.90) in those with longer diabetes duration. The cumulative incidence of pancreatic cysts in each group was calculated as 1 minus the Kaplan-Meier estimate (Figure 2). Among the 31 877 patients with PCNs, 1315 (4.1%) were subsequently diagnosed with pancreatic cancer.

**Table 2.** Incidence Rates and Multivariate Hazard Ratios of Pancreatic Cysts According to Diabetes Status

Diabetes status	No. of participants	No. of events	No. of person-years	Incidence per 1000 person-years	Hazard ratio (95% CI)		
					Unadjusted	Adjusted^a^	
						Model 1	Model 2
Normoglycemia	2 645 696	19 390	26 862 277.3	0.72	1 [Reference]	1 [Reference]	1 [Reference]
Impaired fasting glucose	880 842	7923	8 856 331.8	0.89	1.24 (1.21-1.28)	1.05 (1.02-1.08)	1.06 (1.03-1.08)
Shorter diabetes duration (<5 y)	220 004	2684	2 153 171.4	1.25	1.7 (1.67-1.81)	1.22 (1.17-1.3)	1.23 (1.18-1.28)
Longer diabetes duration (≥5 y)	110 134	1880	1 034 975.8	1.82	2.56 (2.44-2.69)	1.37 (1.3-1.44)	1.37 (1.31-1.44)

^a^ Model 1 was adjusted for age, sex, hypertension, dyslipidemia, income status, smoking status, alcohol consumption, regular physical activity, and body mass index. Model 2 was further adjusted for hypertriglyceridemia.

A total of 25 517 of 3 856 676 individuals (0.7% of the total population) were identified as having developed pancreatic cancer during the follow-up period (based on *ICD-10* code C25.x). Furthermore, the proportion of individuals undergoing pancreatectomy was markedly higher among those with pancreatic cysts (3.5%) than in the overall population (0.15%), and pancreatic cancer requiring surgery was also substantially more frequent (1.5% vs 0.06%).

Patients with diabetes had higher risk of PCN than those without diabetes, even after adjusting for confounding factors such as age, sex, BMI, smoking status, alcohol consumption, hypertension, and dyslipidemia. The risk of PCN increased with the deterioration of glycemic status. The adjusted HR (AHR) for PCN was 1.06 (95% CI, 1.03-1.08) in those with IFG and 1.23 (95% CI, 1.18-1.28) in those with a shorter diabetes duration. Patients with a longer diabetes duration had approximately 1.4-fold greater risk of developing pancreatic cysts than participants with normoglycemia (AHR, 1.37 [95% CI, 1.31-1.44]).

### Association of Diabetes With PCN Occurrence by Subgroups

Subgroup analyses were performed to determine whether the risk between diabetes and PCN development differed based on participant demographic characteristics, lifestyle factors, or comorbidities (summarized in Table 3). The AHRs for diabetes with PCN occurrence were significantly higher in individuals younger than 60 years with diabetes than in participants 60 years or older with diabetes (AHR, 1.34 [95% CI, 1.27-1.40] compared with 1.21 [95% CI, 1.16-1.27]; *P* = .003) and in males with diabetes than in females with diabetes (AHR, 1.32 [95% CI, 1.26-1.38] compared with 1.20 [95% CI, 1.15-1.26]; *P* = .005). According to a subgroup analysis of antidiabetic medication use (1 oral medication, ≥2 oral medications, and insulin use), insulin users showed a higher risk of pancreatic cyst occurrence than individuals using oral antidiabetic medications only, particularly among those with a diabetes duration of 5 years or longer (eTable 1 in Supplement 1).

**Table 3.** Multivariate Hazard Ratios of Pancreatic Cyst Risk by Diabetes Status in Subgroups Defined by Demographic Characteristics, Lifestyle Factors, and Comorbidities

Subgroup	No. of individuals	No. of events	No. of person-years	Incidence per 1000 person-years	Hazard ratio (95% CI)		
					Unadjusted	Adjusted^a^	
						Model 1	Model 2
**Age**							
<60 y							
Without diabetes	2 868 073	17 284	29 377 448.4	0.59	1 [Reference]	1 [Reference]	1 [Reference]
With diabetes	181 284	1905	1 822 793.8	1.05	1.78 (1.70-1.87)	1.32 (1.26-1.39)	1.34 (1.27-1.40)
≥60 y							
Without diabetes	658 465	10 029	6 341 160.7	1.58	1 [Reference]	1 [Reference]	1 [Reference]
With diabetes	148 854	2659	1 365 353.4	1.95	1.25 (1.20-1.30)	1.21 (1.16-1.26)	1.21 (1.16-1.27)
*P* value	NA	NA	NA	NA	<.001	.006	.003
**Sex**							
Female							
Without diabetes	1 628 269	15 332	16 583 762.6	0.92	1 [Reference]	1 [Reference]	1 [Reference]
With diabetes	126 461	2213	1 234 855.9	1.79	1.96 (1.87-2.05)	1.20 (1.14-1.25)	1.20 (1.15-1.26)
Male							
Without diabetes	1 898 269	11 981	19 134 846.5	0.63	1 [Reference]	1 [Reference]	1 [Reference]
With diabetes	203 677	2351	1 953 291.3	1.20	1.94 (1.9-2.03)	1.31 (1.25-1.37)	1.32 (1.26-1.38)
*P* value	NA	NA	NA	NA	.85	.004	.005
**Smoking status**							
Never smoker							
Without diabetes	2 118 024	18 664	21 509 354.8	0.87	1 [Reference]	1 [Reference]	1 [Reference]
With diabetes	183 365	2915	1 773 956.2	1.64	1.913 (1.84-1.989)	1.22 (1.17-1.27)	1.22 (1.18-1.28)
Former smoker							
Without diabetes	490 137	3885	4 939 959.0	0.79	1 [Reference]	1 [Reference]	1 [Reference]
With diabetes	60 759	793	585 596.7	1.35	1.74 (1.612-1.878)	1.24 (1.15-1.34)	1.25 (1.16-1.35)
Current smoker							
Without diabetes	918 377	4764	9 269 295.4	0.51	1 [Reference]	1 [Reference]	1 [Reference]
With diabetes	86 014	856	828 594.2	1.03	2.032 (1.89-2.186)	1.39 (1.29-1.49)	1.40 (1.30-1.51)
*P* value	NA	NA	NA	NA	.014	.008	.006
**Alcohol consumption**							
None							
Without diabetes	1 803 356	17 070	18 202 631.4	0.94	1 [Reference]	1 [Reference]	1 [Reference]
With diabetes	187 193	2959	1 789 133.5	1.65	1.78 (1.72-1.86)	1.21 (1.164-1.261)	1.22 (1.17-1.27)
Mild consumption							
Without diabetes	1 428 978	8389	14 545 551.5	0.58	1 [Reference]	1 [Reference]	1 [Reference]
With diabetes	109 336	1273	1 072 881.5	1.19	2.08 (1.96-2.20)	1.38 (1.30-1.46)	1.39 (1.31-1.48)
Heavy consumption							
Without diabetes	294 204	1854	2 970 426.3	0.62	1 [Reference]	1 [Reference]	1 [Reference]
With diabetes	33 609	332	326 132.3	1.02	1.65 (1.46-1.85)	1.17 (1.04-1.31)	1.18 (1.05-1.33)
*P* value	NA	NA	NA	NA	<.001	.001	.001
**Regular physical activity**							
No							
Without diabetes	2 906 699	21 818	29 429 461.2	0.74	1 [Reference]	1 [Reference]	1 [Reference]
With diabetes	258 104	3501	2 485 014.8	1.41	1.92 (1.85-1.99)	1.25 (1.21-1.30)	1.26 (1.21-1.31)
Yes							
Without diabetes	619 839	5495	6 289 147.9	0.87	1 [Reference]	1 [Reference]	1 [Reference]
With diabetes	72 034	1063	703 132.4	1.51	1.75 (1.64-1.86)	1.25 (1.17-1.33)	1.25 (1.17-1.34)
*P* value	NA	NA	NA	NA	.012	.93	.90
**Hypertriglyceridemia**							
No							
Without diabetes	2 425 332	17 738	24 598 664.4	0.72	1 [Reference]	1 [Reference]	1 [Reference]
With diabetes	125 532	1714	1 202 488.5	1.43	2 (1.90-2.10)	1.27 (1.21-1.34)	1.28 (1.21-1.34)
Yes							
Without diabetes	1 101 206	9575	11 119 944.8	0.86	1 [Reference]	1 [Reference]	1 [Reference]
With diabetes	204 606	2850	1 985 658.7	1.44	1.68 (1.61-1.75)	1.24 (1.18-1.29)	1.24 (1.19-1.29)
*P* value	NA	NA	NA	NA	<.001	.39	.36
**CKD**							
No							
Without diabetes	3 302 347	25 076	33 500 863.0	0.75	1 [Reference]	1 [Reference]	1 [Reference]
With diabetes	288 298	3929	2 811 383.5	1.40	1.88 (1.82-1.95)	1.27 (1.22-1.31)	1.27 (1.23-1.32)
Yes							
Without diabetes	224 191	2237	2 217 746.2	1.01	1 [Reference]	1 [Reference]	1 [Reference]
With diabetes	41 840	635	376 763.7	1.69	1.71 (1.57-1.87)	1.18 (1.08-1.29)	1.19 (1.09-1.30)
*P* value	NA	NA	NA	NA	.049	.15	.14

Abbreviations: CKD, chronic kidney disease; NA, not applicable.

^a^ Model 1 was adjusted for age, sex, hypertension, dyslipidemia, income status, smoking status, alcohol consumption, regular physical activity, and body mass index. Model 2 was further adjusted for hypertriglyceridemia.

In terms of smoking habits, the risks for diabetes and PCN occurrence were higher in current smokers than in nonsmokers. Current smokers with diabetes had a 1.4-fold higher risk of developing PCNs than those without diabetes; however, never smokers and former smokers with diabetes had a 1.2-fold higher risk than individuals with normoglycemia (AHR, 1.40 [95% CI, 1.30-1.51] vs 1.22 [95% CI, 1.18-1.28] and 1.25 [95% CI, 1.16-1.35], respectively; *P* = .006). When comparing alcohol consumption status, the association between diabetes and PCN occurrence was greatest in the mild consumption group. Diabetes was not associated with PCN occurrence among patients with hypertriglyceridemia or CKD.

### Additional Analyses

#### Changes in Diabetes Status and Duration During Follow-Up

To account for changes in diabetes status and duration during follow-up, an additional analysis was conducted to assess PCN risk among participants who underwent both the 2009 and 2012 health examinations. Transitions in diabetes status and duration were categorized (eg, normoglycemia to IFG, normoglycemia to diabetes duration <5 years, and diabetes duration <5 years to ≥5 years), and AHRs were estimated for each transition group (eTable 2 in Supplement 1). In this analysis, a stepwise increase in risk of pancreatic cyst occurrence was observed with longer diabetes duration and progression from normoglycemia to diabetes.

#### Aging, Diabetes, and PCN Occurrence During Follow-Up

To evaluate the association of aging with diabetes and PCN occurrence during follow-up, participants who reached age 60 were censored at that point; the association was also found between diabetes and PCN occurrence (AHR, 1.31 [95% CI, 1.23-1.41]). To examine whether preexisting pancreatic cancer was associated with this finding, we performed a sensitivity analysis including preexisting pancreatic cancer at baseline as a covariate. The prevalence of preexisting pancreatic cancer was 0.04% in the total population, and adjustment for this variable did not materially alter the results, with HRs identical up to the third decimal place.

To evaluate possible multicollinearity between age and diabetes duration, we calculated variance inflation factors (VIFs) for all covariates in the multivariable Cox proportional hazards regression model. All VIFs were less than 2.0, confirming model stability (eTable 3 in Supplement 1).

## Discussion

In this nationwide population-based study of Korean adults, diabetes was associated with an increased risk of PCNs. Notably, individuals with a longer diabetes duration (≥5 years) had a 1.4-fold greater risk of PCNs, and diabetes incidence increased progressively with worsening glycemic status (0.72 per 1000 person-years for normoglycemia vs 1.82 per 1000 person-years for longer diabetes duration). These findings suggest that chronic dysregulation of glucose metabolism may be a factor in PCN development.

In particular, the AHRs for diabetes and PCN occurrence were higher in subgroups of younger patients (<60 years), males, and current smokers. Although the overall incidence of pancreatic cysts increased with age and was higher in females, consistent with previous reports of a female predominance of MCNs and SCAs,^23,24^ the relative impact of diabetes was more pronounced in younger individuals and males in this study. This finding may imply differing biological susceptibilities by age and sex and potentially a disproportionate effect of diabetes on the development of IPMNs, which typically exhibit a more balanced sex distribution.

Previous studies have predominantly focused on diabetes as a risk factor for pancreatic cancer across the full spectrum of diseases, such as prediabetes, new-onset diabetes, and long-standing diabetes.^25,26,27^ However, studies investigating the association between diabetes and pancreatic cyst development remain limited. In this context, the present study is notable because it demonstrates an association between diabetes and PCN development through a nationwide population-based study among Korean adults. These associations may be biologically plausible, given that chronic hyperglycemia is thought to promote inflammation and oxidative stress, which could predispose patients to ductal cell injury and subsequent cystic remodeling. In line with this possibility, subgroup analysis based on the type and number of antidiabetic medications used suggested that insulin users had a higher risk of PCN occurrence, particularly those with a longer diabetes duration. This finding suggests that long-term poorly controlled diabetes may further increase the risk of developing PCN.

Chronic inflammation and cell injury in a genetically susceptible ductal epithelium, such as with activating *GNAS* variants (eg, R201H/C), have been implicated in promoting cystic change and neoplastic progression in the pancreas.^28^ *GNAS*-driven signaling can induce ductal dilation, mucin production, and enlarge cystic lesions.^29^ Although the association between hyperglycemia and cystic change in *GNAS*-variant cells has not been clearly established in the existing literature, hyperglycemia can induce changes in pancreatic ductal cell properties.^30,31^ In *KRAS*-variant cells, hyperglycemia has also been shown to enhance STAT3 phosphorylation and MYC expression, promoting tumor progression.^32^ These observations suggest that diabetes may contribute to PCN formation in individuals with genetic susceptibility, such as those with variants in *GNAS*, *KRAS*, or both.

Findings of subgroup analysis further suggested that modifiable risk factors such as smoking and alcohol consumption may also play a role in PCN formation in patients with diabetes. Current smokers with diabetes had a 1.4-fold higher risk of developing PCNs than nonsmokers, and mild alcohol consumption in individuals with diabetes was associated with an even greater risk. These findings highlight the interplay between metabolic and lifestyle-related factors in pancreatic injury and cyst formation. Consistent with our results, prior studies have reported that smoking is associated with both the occurrence and malignant transformation of IPMNs.^33,34,35^ Although these studies primarily focused on the progression of preexisting cystic lesions, the shared biological mechanism whereby smoking promotes chronic pancreatic inflammation and ductal epithelial injury may also contribute to the initiation of pancreatic cyst formation. Our population-based findings therefore extend previous evidence, suggesting that smoking may not only accelerate cyst progression but also facilitate initial cyst development, particularly in individuals with diabetes and metabolic susceptibility.

Considering the increased risk of PCNs in patients with diabetes, especially younger men and smokers, targeted surveillance in these high-risk groups may be warranted. In addition, smoking cessation and improved diabetes management could potentially help reduce cyst formation. However, most pancreatic cysts are indolent, and current guidelines^5^ recommend discontinuing surveillance for low-risk lesions, especially in older adults or those with substantial comorbidities. Therefore, our findings support a focused, risk-stratified approach to cyst surveillance rather than routine intensification of monitoring for all patients with diabetes. Future studies investigating the progression of cystic lesions to clinically relevant tumors, including pancreatic cancer or high-risk IPMNs, are warranted to further refine surveillance strategies.

To further assess the robustness of our findings, several complementary analyses were performed. First, because the NHIS health examination data are collected biennially, a fully time-varying Cox regression model could not be implemented. Instead, we conducted an additional analysis among participants who underwent both the 2009 and 2012 examinations, approximating time-varying transitions in diabetes status and duration. This analysis demonstrated a stepwise increase in pancreatic cyst risk with longer diabetes duration and progression from normoglycemia to diabetes, consistent with the main results. Second, to evaluate the association between aging and diabetes and PCN occurrence during follow-up, participants who reached age 60 were censored at that point, and the finding was consistent (AHR, 1.31 [95% CI, 1.23-1.41]). Third, to further explore this finding, a sensitivity analysis was conducted that included preexisting pancreatic cancer at baseline as a covariate. The prevalence of preexisting pancreatic cancer was only 0.04% in the total population, and adjustment for this variable did not materially alter the results. Finally, to evaluate possible multicollinearity between age and diabetes duration, we calculated VIFs for all covariates in the multivariable model, all of which were less than 2.0, confirming model stability. Although these additional analyses cannot fully substitute time-varying modeling, they collectively support the robustness and internal validity of our findings.

The prevalence of pancreatic cysts in this study was 0.8% in the entire cohort and 1.6% in individuals aged 60 years or older. These rates are lower than those reported in imaging-based studies, where the prevalence ranges from 2% to 38%, particularly among older adults.^36^ This discrepancy likely reflects the methodological rigor: (1) claims data capture clinically relevant cysts rather than incidental findings; (2) a washout period excludes preexisting cases; and (3) a 1-year lag minimizes surveillance bias. These findings are consistent with previous claims-based research using Korean national data, which reported a pancreatic cyst prevalence of 0.23% in 2018, which is substantially lower than imaging-based estimates but aligned with our methodology and case definitions.^37^ These approaches help ensure that the participants included in the analysis represent individuals with newly diagnosed, clinically significant cysts. Moreover, the use of nationwide claims data enables robust population-level inferences while minimizing overestimation driven by increased imaging sensitivity.

In this nationwide cohort study, 0.7% of the total population was identified as having developed pancreatic cancer during the follow-up period (based on *ICD-10* code C25.x). Among the 31 877 individuals who developed a pancreatic cyst, 1315 (4.1%) were subsequently diagnosed with pancreatic cancer. These values were obtained from the same cohort used for the primary analysis and suggest that cystic lesions may precede malignant neoplasms in a subset of individuals. Furthermore, the proportion of individuals undergoing pancreatectomy was markedly higher among those with pancreatic cysts (3.5%) than in the overall population (0.15%), and pancreatic cancer requiring surgery was also substantially more frequent (1.5% vs 0.06%). These findings suggest that cystic lesions, although relatively uncommon, are more frequently linked to surgically treated or malignant pancreatic disease compared with the general population. However, the absolute incidence remained low, and most cysts were likely to be indolent. Because the NHIS claims database lacks detailed information on surgical indications and histopathologic findings, we were unable to determine whether these cases represented clinically relevant tumors such as IPMNs requiring resection. These findings underscore the need for cautious interpretation: while the presence of a cyst may signal an elevated malignant potential in some cases, population-wide surveillance of all cystic lesions, particularly among patients with diabetes (a highly prevalent condition), may not be justified. Future studies integrating imaging, histopathologic, and longitudinal data are warranted to identify cyst subtypes or patient profiles that confer a clinically meaningful risk of malignant neoplasms.

### Limitations

This study has several limitations. First, although the use of a nationwide population-based database offers broad generalizability, it also introduces limitations inherent to claim-based databases. These limitations include potential misclassification due to reliance on *ICD-10* codes, which precludes accurate differentiation among PCN subtypes (eg, IPMNs, MCNs, and SCAs) and prevents identification of very small, indeterminate cysts, as well as limited clinical granularity, such as a lack of data on laboratory values, lifestyle factors, or family history. Second, the absence of detailed imaging information, including cyst size, location, mural nodules, and main pancreatic duct dilation, restricted the assessment of key radiologic features that may affect cyst behavior and malignant potential. The diagnostic modality for pancreatic cysts was not available in the claims data; however, in clinical practice, most lesions are confirmed by cross-sectional imaging, such as computed tomography (CT) or magnetic resonance imaging (MRI), before an *ICD-10* code is assigned, whether initially detected on ultrasonography or directly identified on CT or MRI. Thus, while most identified cysts likely reflect confirmed radiologic diagnoses, undetected cysts, particularly small or tail-located lesions that may be missed on ultrasonography, could have led to an underestimation of the true prevalence. We explicitly note that due to these limitations, our findings reflect the overall risk of pancreatic cyst development rather than subtype-specific risks. Future studies with imaging- or pathology-based data are needed to better assess subtype-specific risks and malignant potential of pancreatic cystic lesions in patients with diabetes. Third, the claims database structure precludes reliable determination of the temporal association between incident PCNs and subsequent development of diabetes, preventing evaluation of potential type 3c diabetes risk. Moreover, because participants did not undergo standardized or routine follow-up imaging, and most cysts were incidentally detected during evaluations conducted for other clinical indications, there may be heterogeneity in diagnostic timing and methods. Finally, variability in diagnostic and coding practices across institutions, along with the potential immortal time bias inherent in retrospective cohort designs, may further affect the validity of the findings and limit causal interpretation.

## Conclusions

In this cohort study of 3 856 676 Korean adults, longer diabetes duration was associated with a modestly increased risk of PCNs after adjustment for major confounding factors, particularly among younger individuals, male individuals, and current smokers. Although these findings do not establish causality, they suggest that metabolic dysregulation may be a factor in pancreatic cyst development. Despite the inherent limitations of the claims-based design, this study provides valuable epidemiologic evidence linking diabetes to PCNs in a large nationwide population. Health care professionals should consider these factors, especially when evaluating pancreatic findings in patients with diabetes and additional risk factors. Further prospective studies incorporating imaging and molecular data are warranted to clarify the clinical significance and biological mechanisms underlying this association.

## Notes

Supplement 1. **eTable 1.** Subgroup Analyses Based on the Type and Number of Antidiabetes Medications Taken by Patients With Diabetes **eTable 2.** Risk of Pancreatic Cystic Neoplasms According to Transitions in Diabetes Status and Duration (Between 2009 and 2012) **eTable 3.** Variance Inflation Factor Values for Covariates in the Multivariable Cox Proportional Hazards Regression Model

Supplement 2.
Data Sharing Statement
