# Supplementary material for: Pancreatic Cystic Neoplasm Risk Among Individuals With Diabetes
Source: JAMA Netw Open. 2026 Feb 13;9(2):e2556951. doi: 10.1001/jamanetworkopen.2025.56951 (PMC12905656; doi:10.1001/jamanetworkopen.2025.56951)
Supplement: Supplement 1. — eTable 1. Subgroup Analyses Based on the Type and Number of Antidiabetes Medications Taken by Patients With Diabetes eTable 2. Risk of Pancreatic Cystic Neoplasms According to Transitions in Diabetes Status and Duration (Between 2009 and 2012) eTable 3. Variance Inflation Factor Values for Covariates in the Multivariable Cox Proportional Hazards Regression Model [file jamanetwopen-e2556951-s001.pdf]

# Supplemental Online Content

Cho IR, Chang SH, Lee SH, et al. Pancreatic cystic neoplasm risk among individuals with diabetes. *JAMA Netw Open*. 2026;9(2):e2556951.
doi:10.1001/jamanetworkopen.2025.56951

**eTable 1.** Subgroup Analyses Based on the Type and Number of Antidiabetes Medications Taken by Patients With Diabetes

**eTable 2.** Risk of Pancreatic Cystic Neoplasms According to Transitions in Diabetes Status and Duration (Between 2009 and 2012)

**eTable 3.** Variance Inflation Factor Values for Covariates in the Multivariable Cox Proportional Hazards Regression Model

This supplemental material has been provided by the authors to give readers additional information about their work.

© 2026 Cho IR et al. *JAMA Network Open*.

**eTable 1.** Subgroup Analyses Based on the Type and Number of Antidiabetes Medications Taken by Patients With Diabetes

| | n | Events (n) | PYs | Incidence per 1,000 PY | HR (95% CI) | | |
|---|---|---|---|---|---|---|---|
| | | | | | Unadjusted | Adjusted - Model1 | Adjusted - Model2 |
| ***All DM patients*** | | | | | | | |
| 0 oral medication | 132115 | 1261 | 1304543.35 | 0.97 | 1 (Ref.) | 1 (Ref.) | 1 (Ref.) |
| 1 oral medication | 50924 | 842 | 490642.77 | 1.72 | 1.782 (1.633,1.945) | 1.33 (1.214,1.457) | 1.313 (1.199,1.439) |
| ≥2 oral medication | 120260 | 1974 | 1152910.43 | 1.71 | 1.781 (1.66,1.911) | 1.381 (1.281,1.488) | 1.365 (1.267,1.471) |
| Insulin use | 26839 | 487 | 240050.64 | 2.03 | 2.139 (1.927,2.375) | 1.591 (1.427,1.773) | 1.567 (1.405,1.747) |
| ***DM duration <5 years.*** | | | | | | | |
| 0 oral medication | 126667 | 1199 | 1251792.62 | 0.96 | 1 (Ref.) | 1 (Ref.) | 1 (Ref.) |
| 1 oral medication | 35119 | 583 | 341079.5 | 1.71 | 1.788 (1.619,1.974) | 1.349 (1.217,1.497) | 1.338 (1.206,1.484) |
| ≥2 oral medication | 50351 | 783 | 488752.47 | 1.60 | 1.677 (1.532,1.835) | 1.364 (1.242,1.498) | 1.353 (1.232,1.486) |
| Insulin use | 7867 | 119 | 71546.82 | 1.66 | 1.763 (1.46,2.128) | 1.382 (1.142,1.673) | 1.369 (1.131,1.657) |
| ***DM duration ≥5 years*** | | | | | | | |
| 0 oral medication | 5448 | 62 | 52750.73 | 1.18 | 1 (Ref.) | 1 (Ref.) | 1 (Ref.) |
| 1 oral medication | 15805 | 259 | 149563.28 | 1.73 | 1.482 (1.123,1.955) | 1.093 (0.824,1.45) | 1.076 (0.811,1.427) |
| ≥2 oral medication | 69909 | 1191 | 664157.96 | 1.79 | 1.533 (1.188,1.979) | 1.18 (0.91,1.531) | 1.167 (0.899,1.514) |
| Insulin use | 18972 | 368 | 168503.81 | 2.18 | 1.89 (1.444,2.473) | 1.414 (1.074,1.861) | 1.389 (1.055,1.829) |

* Abbreviations: DM, diabetes mellitus; HR, hazard ratio; PY, person-year

** (i) Model 1 was adjusted for age, sex, hypertension, dyslipidemia, income status, smoking, alcohol consumption, regular physical activity, and body mass index and (ii) Model 2 was further adjusted for hypertriglyceridemia

© 2026 Cho IR et al. *JAMA Network Open*.

**eTable 2.** Risk of Pancreatic Cystic Neoplasms According to Transitions in Diabetes Status and Duration (Between 2009 and 2012)

| Diabetes status (2009 → 2012) | n | Events (n) | PYs | Incidence per 1,000 PY | HR(95% CI) | | |
|---|---|---|---|---|---|---|---|
| | | | | | Unadjusted | Adjusted - Model 1 | Adjusted - Model 2 |
| Normal → Normal | 905,719 | 4,424 | 6,642,812.33 | 0.67 | 1 (Ref.) | 1 (Ref.) | 1 (Ref.) |
| Normal → IFG | 214,639 | 1,254 | 1,563,112.4 | 0.80 | 1.209 (1.135,1.287) | 1.059 (0.994,1.129) | 1.065 (0.999,1.135) |
| Normal → DM <5 years | 18,682 | 138 | 134,610.96 | 1.03 | 1.545 (1.305,1.831) | 1.137 (0.958,1.348) | 1.150 (0.969,1.364) |
| IFG → IFG | 144,545 | 890 | 1,051,832.33 | 0.85 | 1.274 (1.186,1.37) | 1.000 (0.929,1.077) | 1.007 (0.935,1.084) |
| IFG → DM <5 years | 30,419 | 267 | 219,461.9 | 1.22 | 1.835 (1.622,2.077 | 1.245 (1.098,1.411) | 1.260 (1.111,1.429) |
| DM <5 years → DM <5 years | 60,239 | 459 | 434,787.49 | 1.06 | 1.590 (1.444,1.75) | 1.098 (0.995,1.212) | 1.108 (1.004,1.222) |
| DM <5 years → DM ≥ 5 years | 16,912 | 192 | 120,439.99 | 1.59 | 2.408 (2.084,2.782) | 1.305 (1.126,1.511) | 1.312 (1.132,1.52) |
| DM 5 years → DM ≥5 years | 27,910 | 368 | 196,537.15 | 1.87 | 2.829 (2.544,3.146) | 1.323 (1.184,1.477) | 1.328 (1.189,1.483) |

* Abbreviations: IFG, impaired fasting glucose; DM, diabetes mellitus; HR, hazard ratio; PY, person-year

** (i) Model 1 was adjusted for age, sex, hypertension, dyslipidemia, income status, smoking, alcohol consumption, regular physical activity, and body mass index and (ii) Model 2 was further adjusted for hypertriglyceridemia

© 2026 Cho IR et al. *JAMA Network Open*.

**eTable 3**. Variance Inflation Factor Values for Covariates in the Multivariable Cox Proportional Hazards Regression Model

| Variables | VIF |
|---|---|
| Age | 1.42 |
| Sex | 1.77 |
| BMI | 1.18 |
| Hypertension | 1.36 |
| Dislipidemia | 1.18 |
| Hypertriglyceridemia | 1.21 |
| Smoking | 1.70 |
| Drinking | 1.32 |
| Regular exercise | 1.01 |
| Diabetes duration | 1.18 |

* Abbreviations: VIF, variance inflation factor

© 2026 Cho IR et al. *JAMA Network Open*.
