# Supplementary material for: Pancreatic Cystic Neoplasm Risk Among Individuals With Diabetes
Source: JAMA Netw Open. 2026 Feb 13;9(2):e2556951. doi: 10.1001/jamanetworkopen.2025.56951 (PMC12905656; doi:10.1001/jamanetworkopen.2025.56951)
Supplement: Supplement 2. — Data Sharing Statement [file jamanetwopen-e2556951-s002.pdf]

# Data Sharing Statement

Cho. Pancreatic Cystic Neoplasm Risk Among Individuals With Diabetes. *JAMA Netw Open*. Published February 13, 2026. doi:10.1001/jamanetworkopen.2025.56951

**Data**

**Data available:** The data used in this study are available from the Korean National Health Insurance Service (NHIS), but restrictions apply to their availability. Qualified researchers may request access to the data through official application to the NHIS. Deidentified individual participant data underlying the results reported in this article may be shared by the corresponding author upon reasonable request from investigators with approved research protocols and appropriate data use agreements.
